# Duck plague virus tegument protein vp22 plays a key role in the secondary envelopment and cell-to-cell spread

**DOI:** 10.1186/s13567-023-01191-9

**Published:** 2023-07-17

**Authors:** Liping Wu, Mingshu Wang, Anchun Cheng, Bin Tian, Juan Huang, Ying Wu, Qiao Yang, Xumin Ou, Di Sun, Shaqiu Zhang, Xinxin Zhao, Qun Gao, Yu He, Dekang Zhu, Shun Chen, Mafeng Liu, Renyong Jia

**Affiliations:** 1 Institute of Veterinary Medicine and Immunology, College of Veterinary Medicine, Sichuan Agricultural University, Wenjiang, Chengdu City, 611130 Sichuan China; 2 Key Laboratory of Animal Disease and Human Health of Sichuan Province, Sichuan Agricultural University, Wenjiang, Chengdu City, 611130 Sichuan China; 3 Avian Disease Research Center, College of Veterinary Medicine, Sichuan Agricultural University, Wenjiang, Chengdu City, 611130 Sichuan China; 4 Engineering Research Center of Southwest Animal Disease Prevention and Control Technology, Ministry of Education of the People’s Republic of China, Sichuan Agricultural University, Wenjiang, Chengdu City, 611130 Sichuan China

**Keywords:** Duck plague virus, UL49, VP22, envelopment, cell-to-cell spread

## Abstract

Duck plague virus (DPV) is one of the major infectious and fatal diseases of geese, ducks, and other wild waterfowl. The DPV UL49 gene product VP22 is one of the most abundant tegument proteins. However, the role of the DPV VP22 is enigmatic to be clarified. In this study, we found deletion of the UL49 gene resulted in reduced viral growth curve and smaller plaque size in duck embryo fibroblast (DEF) cells, confirming that DPV VP22 is required for efficient viral growth in vitro. In addition, deletion of the UL49 gene inhibited the secondary envelopment of the virus, the release of viral particles, and the spread of viruses between cells. Our study signified the importance of VP22 for DPV secondary envelopment, release, cell-to-cell spread, and accumulation of viral RNA. These findings provide a basis for further study of the function of VP22 in DPV or other herpesviruses.

**Supplementary Information:**

The online version contains supplementary material available at 10.1186/s13567-023-01191-9.

## Introduction

Herpesviruses are a group of double-stranded DNA viruses with envelope and the tendency of latent infection, which threaten the health of human and animals seriously [1]. The Herpesviridae are composed of three subfamilies of *Alphaherpesvirinae*, *Betaherpesvirinae*, and *Gammaherpesvirinae* [2]. The *Alphaherpesvirinae* subfamily mainly includes Herpes simplex virus type 1 and 2 (HSV-1 and HSV-2, respectively) and Varicella-zoster virus (VZV), which can infect humans. It also includes pseudorabies virus (PRV), Marek’s disease virus (MDV), and duck enteritis virus (DEV), which can infect animals and cause severe morbidity and mortality [3, 4].

Duck plague (DP), also known as duck virus enteritis (DVE), is a sharp and feverish infectious disease caused by duck plague virus (DPV) [5]. The main features of the disease are swelling of the head and neck, high fever, tears, gray-green feces, and the disease has a high incidence and mortality [6, 7]. The herpesvirus particles are spherical and consist of the tegument, a capsid, an outer envelope, and a core containing linear double-stranded DNA [8]. The DPV genome is approximately 160 kb in length and consists of four regions: a unique long region (UL), a unique short region (US), a terminal inverted repeats (TR), and an internal inverted repeats (IR), thus forming the UL-IRS-US-TRS genome structure [9]. The DPV genome contains 78 open reading frames (ORFs), including 65 ORFs in the UL region and 11 ORFs in the US region. The remaining ORFs are located entirely in the IRS and TRS regions [10].

VP22 is a late tegument protein encoded by the UL49 gene, which is abundant in viral particles, and conserved only in the subfamily of alpha-herpesviruses [11, 12]. VP22 interacts with many cellular and viral partners. VZV ORF9p (the analogue of HSV-1 VP22) can interact with AP-1 and can prevent the secondary envelope of VZV by disrupting the binding of ORF9p to AP-1 in infected cells, thereby disrupting the growth of VZV [13]. HSV-1 VP22 interacts with MT in the cytoskeleton and induces the formation of thick MT bundles with high stability and high acetylation [14]. HSV-1 VP22 promotes viral replication and enhances neurotoxicity by regulating the localization and expression of various viral and cellular proteins [15]. Meanwhile, the formation of the optimal gE-VP22-gM-gI-ICP0 complex in the HSV-1 is associated with the efficient morphogenesis and propagation of the virus [16]. VP22, VP16, and VHS form a VP22-VP16-VHS-like complex. Early studies demonstrated that VP16 and VP22 promote mRNA translation rather than degradation by VHS [17]. Later studies confirmed that VP22, but not VP16-VP22 complex, plays an important role in the translation of VHS during infection, and that VP22 regulates VHS activity by specifically rescuing the cytoplasmic localization of late transcripts [18, 19]. The loss of HSV-1 UL49 resulting in reduced translation efficiency can be complemented by secondary mutations in VHS [20]. Furthermore, over-expression of VP22 significantly arrest the cell cycle in S phase and promote the replication of MDV, thereby VP22 can be employed as a viral factor involved in the regulation of cell cycle during MDV infection [21].

The full length of the DPV UL49 gene is 759 bp, and the tegument protein VP22 encoded by the UL49 gene is conserved in alpha-herpesviruses, and has been minimally studied [10]. In this study, to identify the role of the UL49 gene in the DPV life cycle, we constructed and rescued the BAC-CHv-ΔUL49 and BAC-CHv-UL49R viruses by the Red recombination system using the bacterial artificial chromosome (BAC) platform. We found that VP22 is necessary for the efficient growth of DPV in cultured cells, but not essential for viral replication. Deletion of VP22 significantly impaired the secondary envelopment, release, and cell-to-cell spread of the virus. Furthermore, we also found that the UL49 gene affects viral RNA accumulation.

## Materials and methods

### Cells, virus stains, and primers

DEF cells were prepared from 9-day-old duck embryos and cultured in Dulbecco’s modified Eagle’s medium (DMEM) (Gibco Life Technologies, Shanghai, China) supplemented with 10% newborn bovine serum (NBS; Gibco, USA) at a 37 °C incubator with 5% carbon dioxide. The BAC-CHv virus strain was obtained from our laboratory. All of the primers used in this study are listed in Additional file 1.

### Construction and identification of recombinant pBACs

GS1783-pBAC-DPV was obtained from our laboratory, and the entire DPV CHv genome (GenBank: JQ647509.1) was cloned into BAC to generate pBAC-CHv plasmid, which was transformed into a BAC target for bacteria DPV modification. Recombinant pBAC-CHv-ΔUL49 and pBAC-CHv-UL49R were constructed based on the scarless red recombination system of *Escherichia coli* GS1783 [22]. In brief, we amplified a linear PCR product by primers flanked by a 40 bp upstream homologous arm (from the left side of the UL49 gene) and an 80 bp downstream homologous arm (from the left and right side of the UL49 gene) with a kanamycin (Kan) box containing an I-SceI homing endonuclease site in the middle of the product. The Kan resistance gene replaced the UL49 gene in the first homologous recombination. Subsequently, the I-Scel site can cleave through a second homologous recombination to remove the Kan resistance gene. The positive plasmid was extracted using the QIAGEN Plasmid Midi kit (QIAGEN, Germany) for RFLP analysis. The 25 µL system consists of 2 µg plasmid, 4 µL 10× Q.Cut G.Buffer, 2 µL restriction endonuclease Hind III or EcoR I 2 µL, and ddH_2_O consisting of 25 µL. After enzymatic digestion at 37 ℃ for 3 h, the system is subjected to 2–3 h post-infection (hpi) electrophoresis by 1% agarose-gel at 50 V.

### Rescue and identification of recombinant viruses

To obtain the mutated virus, we extracted the recombinant positive plasmid and transfected it into DEF cells using Hieff TransTM liposome transfection reagent (Yeasen, Shanghai, China). After the cells were incubated at 37 ℃ for 6 h, the supernatant was discarded and replaced with fresh medium containing 1% fetal bovine serum (FBS; Invitrogen, USA) until many green fluorescent spots were produced. Cell supernatant was collected (avoid freezing storage during the period), inoculated into new DEF monolayer cells, and passed at least 3 times. The virus was identified by PCR and Western blot.

### Viral multi-step growth curve

BAC-CHv, BAC-CHv-ΔUL49, and BAC-CHv-UL49R recombinant viruses were inoculated with a multiplicity of infection (MOI) of 0.01 in the DEF monolayer of 24-well plates, respectively. Incubated at 37 °C for 1 h, replaced with DMEM containing 1% FBS. Cell and supernatant samples were collected at 24, 48, 72, 96 and 120 hpi after infection. After washing cells with PBS 3 times, 0.5 mL/sample DMEM was added to each well to collect infected cells as cell samples. The supernatant and cellular viral titers were measured by 50% tissue culture infection dose (TCID_50_).

### Viral adsorption, invasion, replication, and release assays

Adsorption: The DEF monolayer cells were placed at 4 °C for 1 h, then 0.001 MOI recombinant virus was inoculated into the cells and incubated at 4 °C for 2 h. The cell surface was washed five times with precooled PBS, replaced with 1.5% methylcellulose (Solarbio, Beijing, China), and cultured in a 37 °C incubator containing 5% carbon dioxide for 48–72 h.

Invasion: The DEF monolayer cells were placed at 4 °C for 1 h, then inoculated with an MOI of 0.001 recombinant viruses and incubated at 4 °C for 2 h, the mixture was removed, then added fresh medium containing 1% FBS and incubated at 37 °C for 2 h, Subsequently replaced with 1.5% methylcellulose and cultured in a 37 °C incubator containing 5% carbon dioxide for 48–72 h.

Replication: DEF cells were incubated with 0.1 MOI of DPV recombinant virus for 6 h, removed the viral mixture, and added fresh medium containing 1% FBS. Cell samples were harvested at 7, 8, 9, and 10 hpi for genomic DNA extraction, followed by quantifying DPV genomic copy number by qRT-PCR.

Release: DEF cells were incubated with 0.01 MOI of DPV recombinant virus for 2 h, washed with PBS 3 times, added 1% FBS medium, and cultured for 16 h. The medium was removed, washed three times with PBS, and replaced with fresh medium containing 1% FBS, and the cell culture supernatant was collected after 15, 30, 45, and 60 min, and then the TCID_50_ was determined.

### Electron microscopy analysis of recombinant viruses

DEF cells were infected with BAC-CHv, BAC-CHv-ΔUL49, or BAC-CHv-UL49R at an MOI of 2. Thirty hours after infection, cells were collected by scraping, centrifuged at 3500 rpm for 10 min, and fixed in 2.5% glutaraldehyde fixative overnight at 4 °C.

At 30 h post-infection, collected by scraping, centrifuged at 3500 rpm for 10 min, and fixed overnight at 4 °C with 2.5% glutaraldehyde fixative solution. All samples were sent to Chengdu Lilai Biotechnology Co., Ltd. for analysis by transmission electron microscopy (Hitachi H-7650, Tokyo, Japan).

### The plaque morphology of recombinant viruses

The 0.005 MOI virus of BAC-CHv, BAC-CHv-ΔUL49 and BAC-CHv-UL49R and DEF cells were incubated at 37 °C for 2 h. The virus incubation solution was replaced with medium containing 1.5% methylcellulose (Solarbio, Beijing, China). Virus plaques were observed by fluorescence microscope (Nikon TI-SR, Japan) at 48 hpi. Thirty green fluorescent plaques from each strain were randomly selected for imaging, and the average plaque size was measured using Image-Pro Plus software (Bio-Rad, California, USA).

### RNA extraction and real-time quantitative PCR (RT-qPCR)

Total RNA was extracted from the infection cell samples using RNAiso Plus (Takara, Japan) and reverse transcription was performed. RT-qPCR was mainly based on the previous methods. The relative levels of gene expression were determined with the 2^−ΔCt^ method.

### Statistical analysis

To determine statistical significance of between the different groups, the student’s t-test or one-way ANOVA was used followed by Tukey’s post hoc test (GraphPad Prism 9 software). All experiments were repeated at least three times. Data are expressed as the mean and standard error of the mean (SEM). Results with *P < 0.05 were considered statistically significant.

## Results

### Construction and identification of recombinant viruses

**Figure 1:**
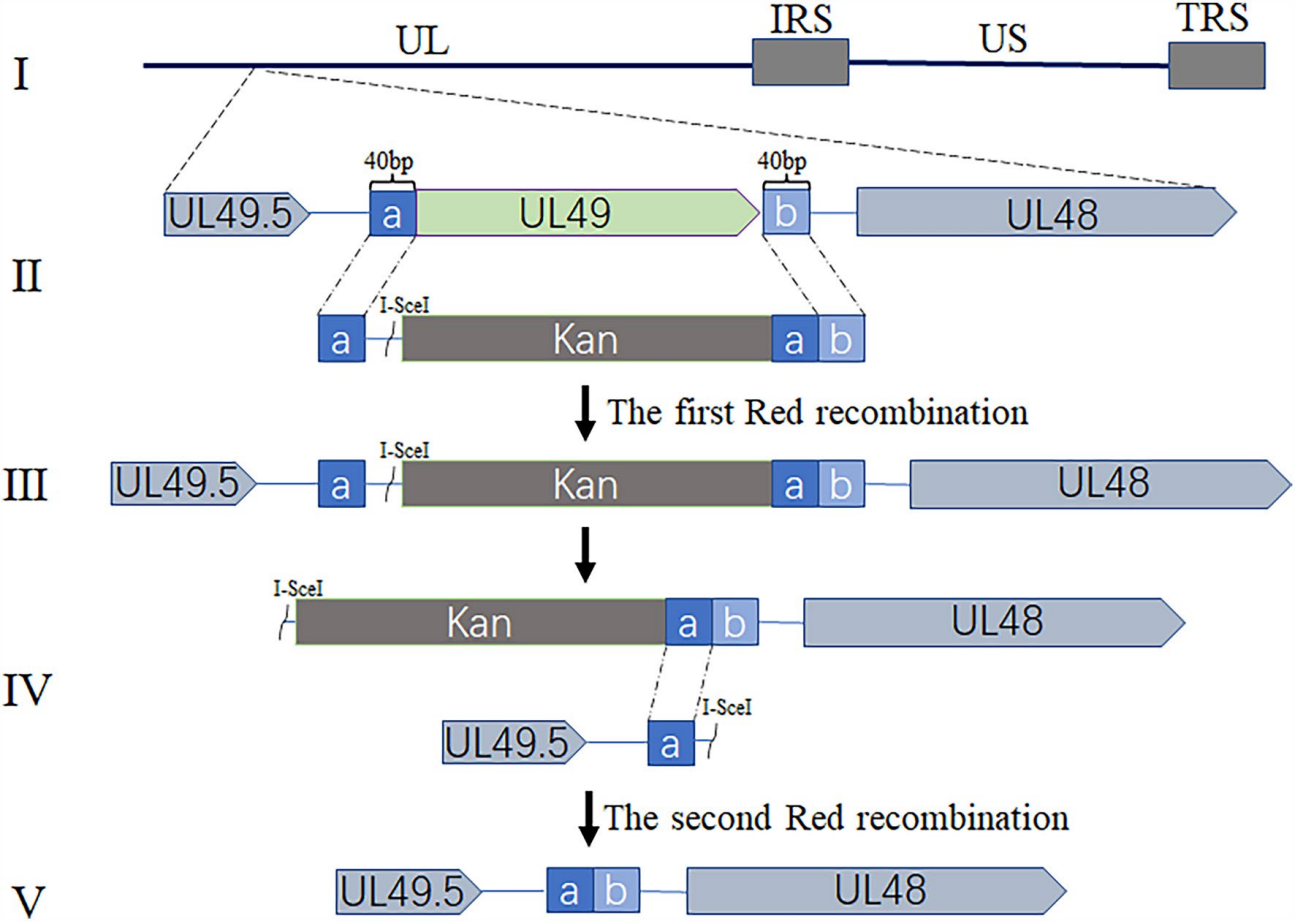
** A schematic diagram of the construction of the BAC-CHv-ΔUL49 virus on the BAC platform.** (I) The genome structure of DPV CHv. (II) The first Red recombination, the UL49 gene was replaced by the Kan resistance gene. (III) The product after the first Red recombination. (IV) The second Red recombination, the I- SecI cleavage site was recognized by the recombinase, and the Kan fragment was removed. (V) The genome structure of BAC-CHv-ΔUL49.

**Figure 2:**
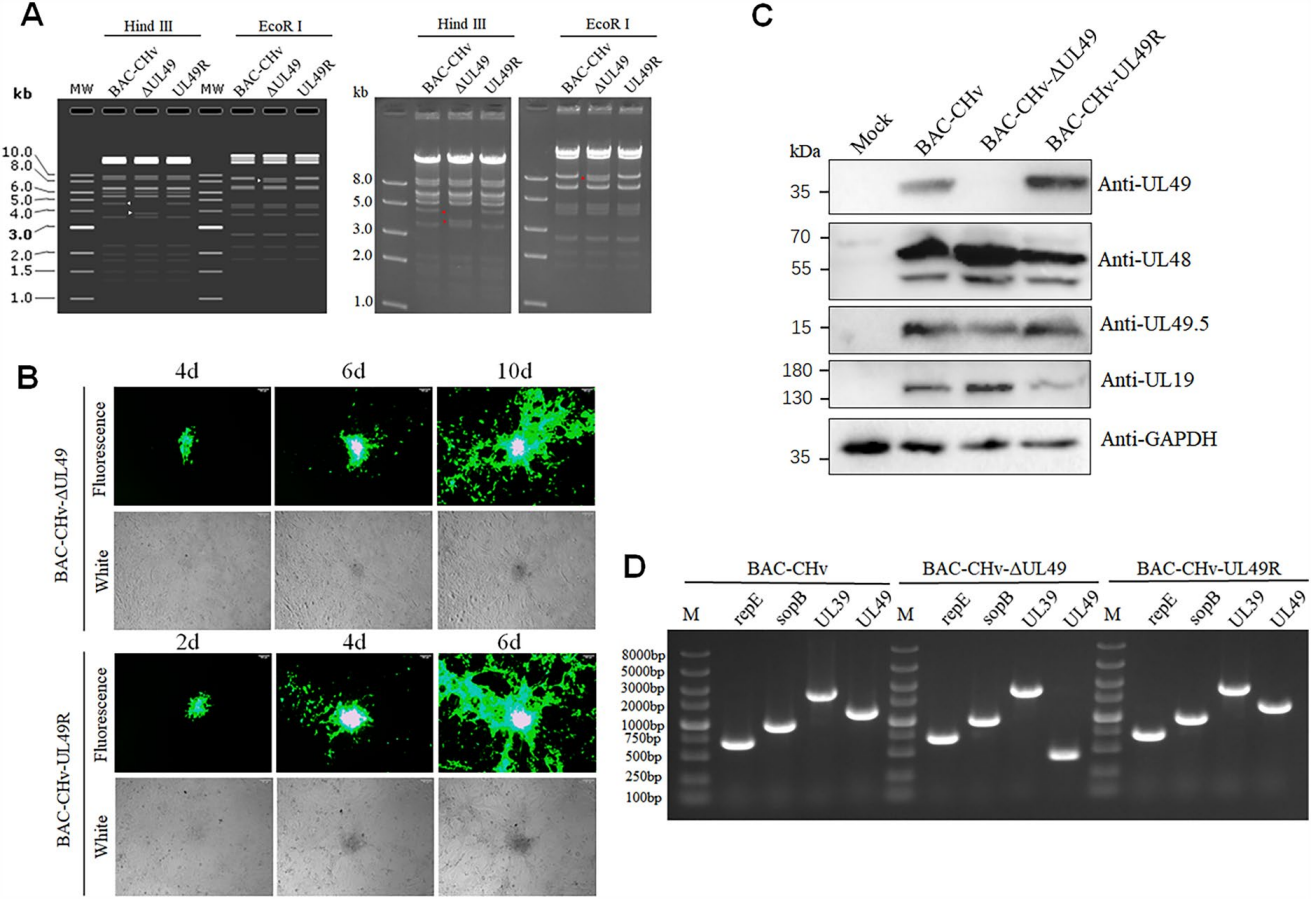
**Rescue and identification of recombinant viruses.** **A** RFLP analysis. The extracted plasmids were cut with restriction enzyme Hind III or EcoR I (The left diagram shows the expected enzyme digestion results, and the right diagram shows the experimental enzyme digestion results). **B** The rescue of UL49 mutant virus. The extracted positive clone plasmid was transfected into DEF cells. The expression of the recombinant viral fluorescence-labeled protein EGFP was observed in DEF cells over time. **C** Western blot analysis of UL49 gene deletion recombinant virus protein expression. **D** Identification of recombinant viruses by PCR.

To investigate the importance of the UL49 gene in the viral life cycle, we constructed a DPV UL49 gene deletion (BAC-CHv-ΔUL49) and a UL49 gene restoration (BAC-CHv-UL49R) infectious clone plasmid using a marker-free two-step Red recombination technique, based on the BAC platform of infectious DPV CHv strain in China (Figure 1). To ensure the correctness of the positive infectious clonal plasmids, we performed restriction fragment length polymorphism (RFLP) analysis (Figure 2A). It is found that the experimental results (right) are consistent with the predicted results (left). Compared with BAC-CHv and UL49R, about 5 kb of bands of ΔUL49 disappeared after digestion by Hind III, with about 4 kb of bands added, and only weak bands appeared at 8 kb after digestion by EcoR I.

To obtain the UL49 gene-related viruses, two positive infectious cloned plasmids with correct detection were transfected into DEF cells, and the changes in the cells were observed at any time. The results are shown in Figure 2B, the green fluorescence was consistent with the cytopathy observed in DEF cells on the second and fourth day after plasmid transfection. The green fluorescence showed a gradually expanding trend. Next, we collected cell supernatant and inoculated it into new DEF cells. After 5 generations of blind passage, cells and supernatant samples were collected. The UL48 gene and UL49.5 gene expression were detected in BAC-CHv, BAC-CHv-ΔUL49 and BAC-CHv-UL49R groups by Western blotting. The expression of the UL49 gene encoding VP22 was detected in BAC-CHv and BAC-CHv-UL49R infected cells, but not in BAC-CHv-ΔUL49 infected and mock cells, indicating that the acquired virus did not express VP22 in the absence of the UL49 gene. It also does not affect the expression of the protein encoded by its neighboring genes (Figure 2C). According to the index-corrected protein abundance index (emPAI) of UL49 in DPV particles detected by mass spectrometry, it can be determined that UL49 is present in a large number of virus particles (Table 1). We extracted the virus DNA from the supernatant samples and identified the extracted virus DNA by PCR. As shown in Figure 2D, BAC original repE (681 bp), sopB (966 bp) were detected in all three groups, while the viral gene UL39 (2433 bp) was detectable, but the entire fragment of the UL49 gene (1195 bp) was only detected in BAC-CHv and BAC-CHv-UL49R groups, while the fragment size after deletion of UL49 gene in the BAC-CHv-ΔUL49 group was 433 bp. Therefore, we determined that the rescue of BAC-CHv-ΔUL49 and BAC-CHv-UL49R virus was successful and can be used in follow-up experiments.

**Table 1:** **Viral content of DPV extracellular virions (partial).**

Protein	Information	Score	Mass	Matches	Sequences	emPAI	NCBI accession
UL19	Capsid protein	647	154774	46 (25)	33 (19)	0.58	AJG04911
UL49	Tegument protein	457	28018	27 (17)	12 (10)	3.83	AJG04879

### VP22 obviously inhibits viral growth curve in vitro

**Figure 3:**
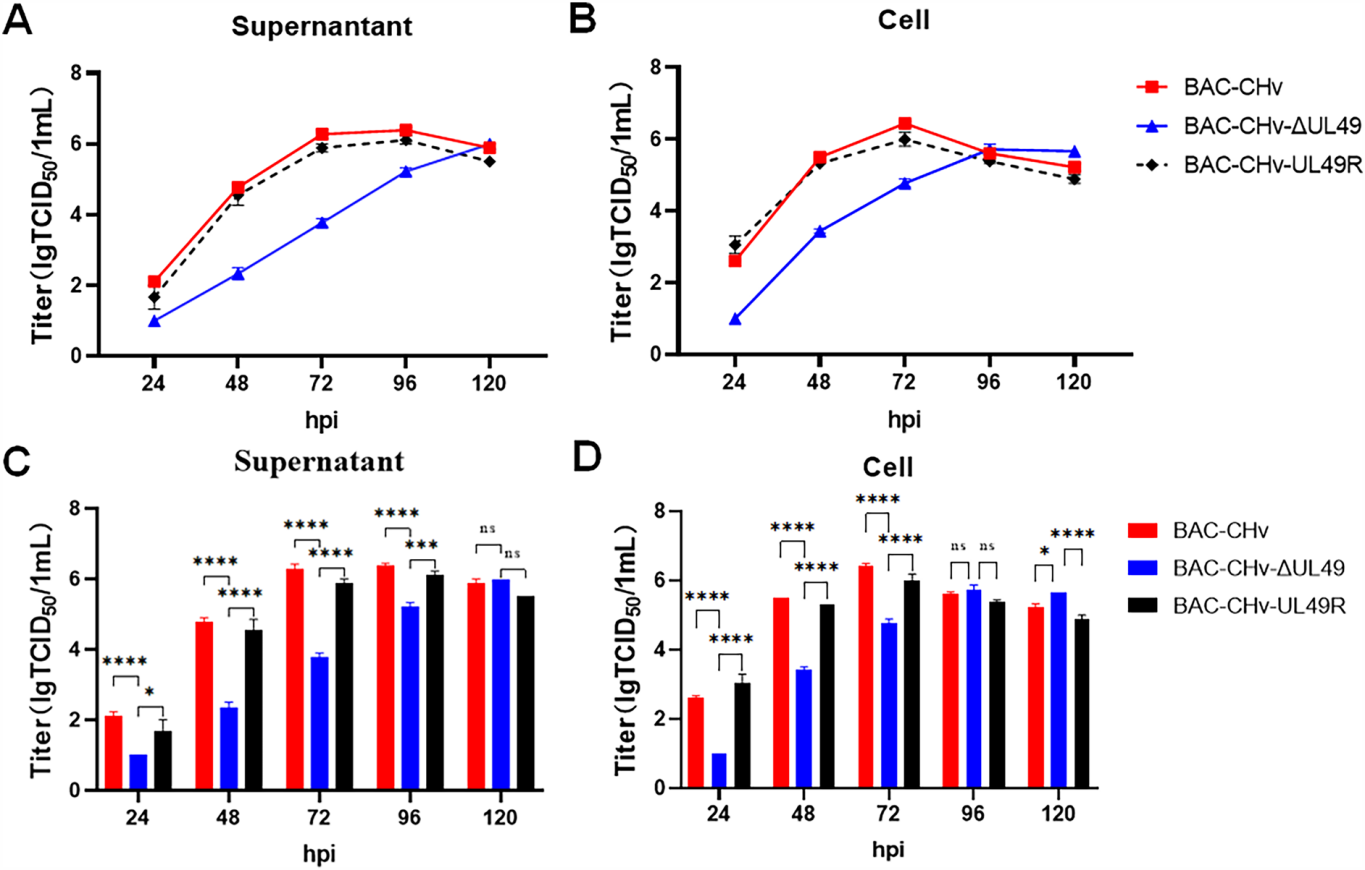
**Multi-step growth curves.** DEF cells in a 24-well plate were infected with BAC-CHv, BAC-CHv-UL49R, and BAC-CHv-ΔUL49 at an MOI of 0.01. The supernatant and cell samples were collected at the specified time points, respectively, and viral titers were measured. Data were calculated as mean ± standard deviation (SD, *P >* 0.05). **A** Virus titer of supernatant. **B** Virus titer of cells. **C** Statistical analysis of virus titer difference in the supernatant at each time point. **D** Statistical analysis of differences in cell virus titers at each time point. The asterisk indicates a significant difference compared to the BAC-CHv and BAC-CHv-UL49R viruses (^∗∗∗∗^*P* < 0.0001; ^∗∗∗^*P* < 0.001; ^∗^*P* < 0.05).

To further characterize whether the UL49 gene impact on DPV proliferation in vitro, we inspected the role of UL49 in DPV replication by determining the multi-step growth kinetics of the virus. The DEF cells were infected with BAC-CHv, BAC-CHv-ΔUL49 and BAC-CHv-UL49R viruses with MOI of 0.01, respectively. Cell samples and supernatants were collected at 24,48,72,96 and 120 hpi after infection. BAC-CHv-ΔUL49 decreased approximately 100-fold compared with BAC-CHv during the early viral infection (Figures 3A and B), indicating that significant growth defect of the BAC-CHv-ΔUL49 virus. To confirm the growth defect of the BAC-CHv-ΔUL49 virus by deletion of the UL49 gene, we reintroduced the UL49 gene into BAC-CHv-ΔUL49 to obtain BAC-CHv-UL49R virus. Subsequently, the growth characteristics of BAC-CHv-UL49R and BAC-CHv were compared. The results showed no significant difference between the two strains, indicating that the growth defect of BAC-CHv-ΔUL49 due to deletion of the UL49 gene.

Meanwhile, to better show the growth differences among the three virus strains, we analyzed experimental data of either the supernatant (Figure 3C) or cells (Figure 3D) viruses using One-way ANOVAs followed by Tukey’s post hoc test (GraphPad Prism 9 software). The results showed the supernatant and cell BAC-CHv-ΔUL49 virus in the early stage of infection, the titer of the virus was significantly lower than that of the BAC-CHv-UL49R and BAC-CHv virus. Therefore, we can conclude that the UL49 gene is important for the efficient growth of the virus in cultured cells.

### VP22 is dispensable for virus adsorption, invasion and genome replication

**Figure 4:**
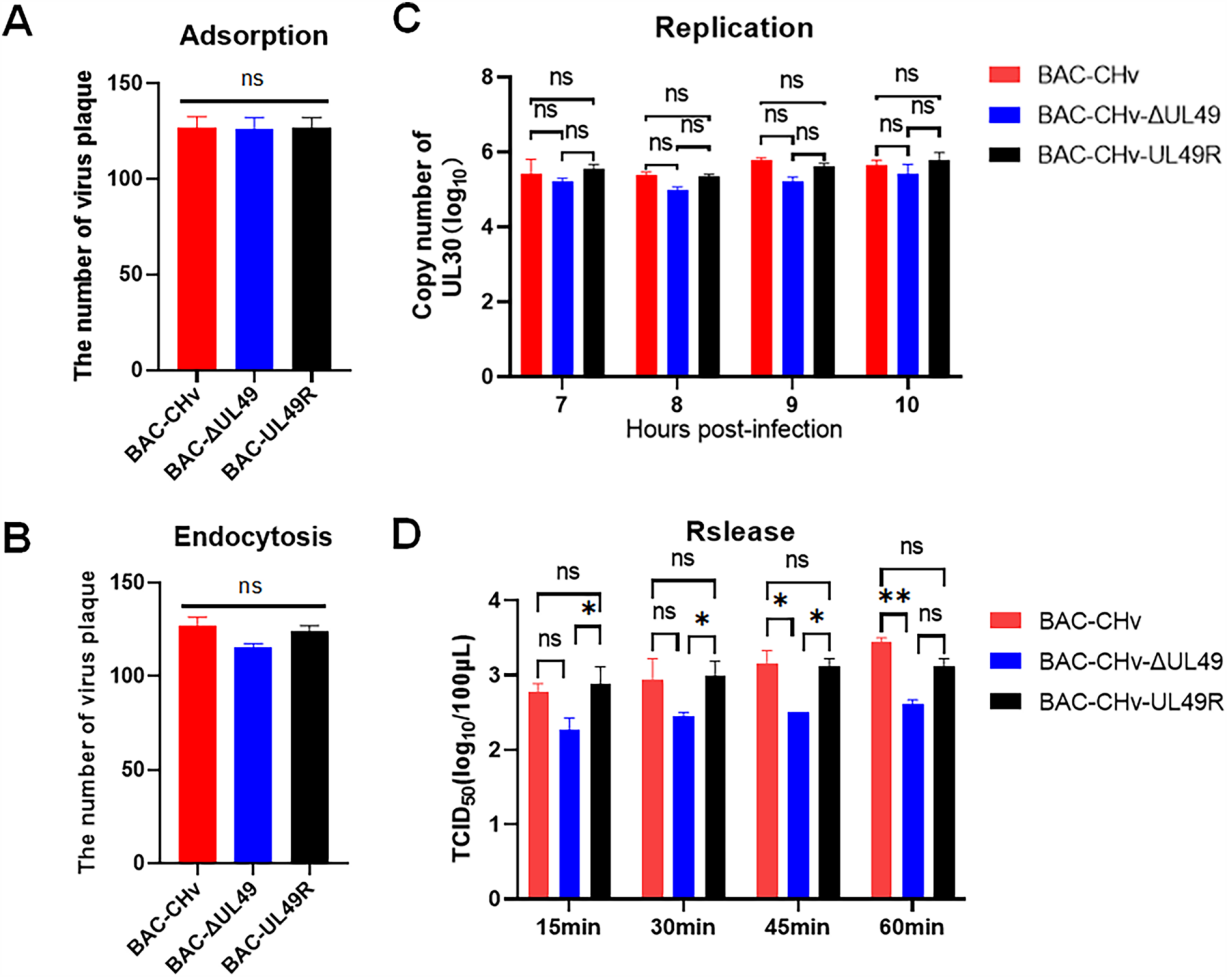
**The effect of UL49 gene on virus adsorption, invasion, replication and release.** The number of plaques in virus-infected cells was counted to determine the effect of the UL49 gene on virus **A** adsorption and **B** endocytosis. DNA was extracted from virus-infected cell samples and the copy number was assayed to determine the effect on **C** virus replication. The effect of the UL49 gene on **D** virus release can be determined from the TCID_50_ of the supernatant source samples (**P < 0.01; ^∗^*P* < 0.05).

To explore the influence of the UL49 gene on the steps of the viral life cycle, we firstly investigated the adsorption (Figure 4A) and invasion (Figure 4B) ability of the UL49 gene deletion virus on the host cell surface. Comparing the number of plaques in the infected cells of the three strains, the date showed the UL49 gene did not change the adsorption and invasion of mature virions on the surface of host cells.

Next, we checked the impact of the UL49 gene on viral genomic DNA replication by infecting DEF cells with three viruses at an MOI of 0.01 and incubating them for 6 h. Infected cells were collected at 7, 8, 9, and 10 hpi. The viral genomic DNA was extracted and the copy number of the virus was detected by RT-qPCR with specific primer to DPV UL30. The results showed no significant difference in the genome content of the three strains (Figure 4C), indicating that deletion of the UL49 gene did not affect the replication of viral genomic DNA. Therefore, these data demonstrated that VP22 is dispensable for virus adsorption, invasion and genome replication.

### VP22 promotes the assembly and release of viral particles

**Figure 5:**
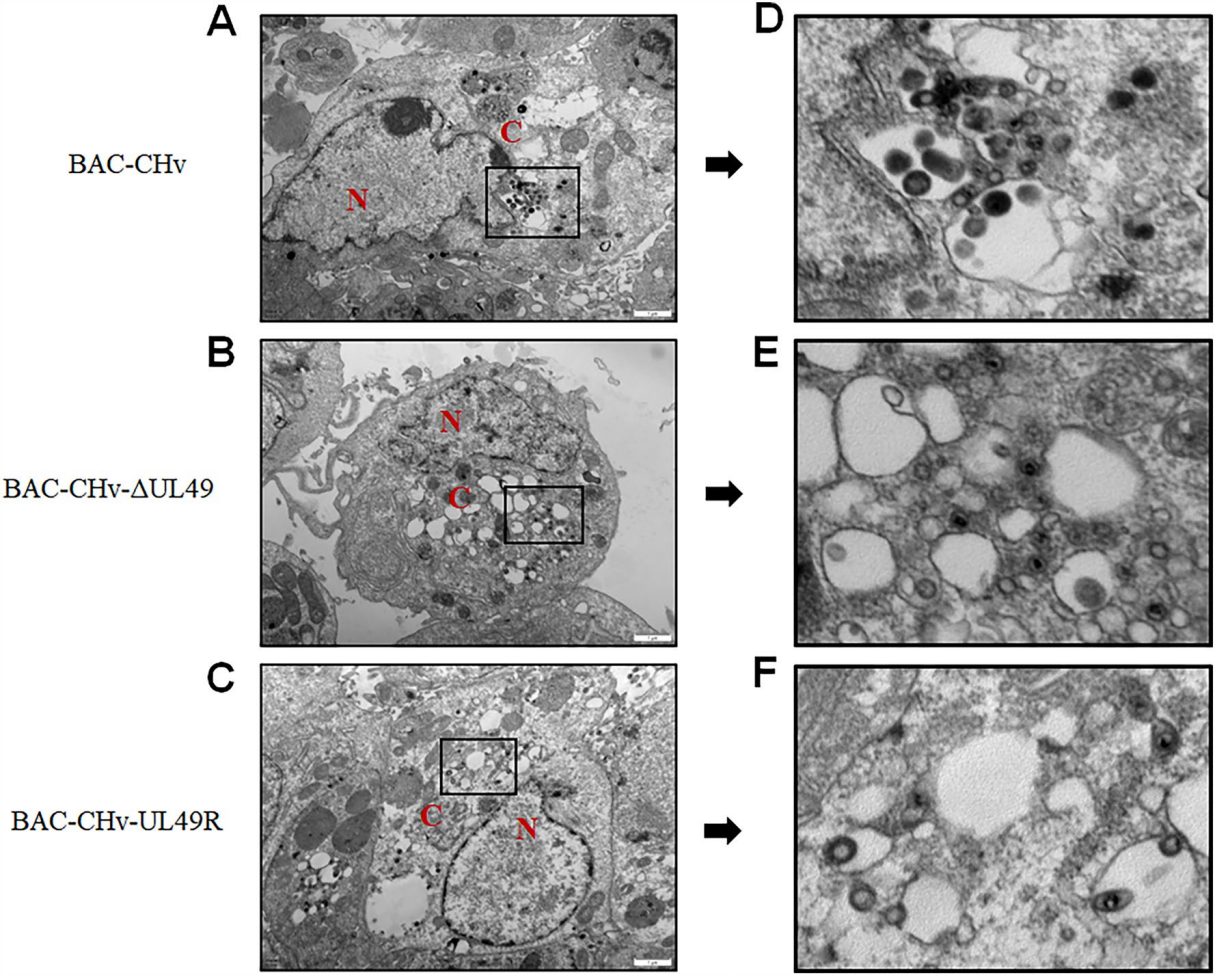
**Electron microscopy analysis BAC-CHv, BAC-CHv-ΔUL49 or BAC-CHv-UL49R infected DEF cells.** **A**–**C** The electron microscopic pictures of DEF cells infected with BAC-CHv, BAC-CHv-ΔUL49, and BAC-CHv-UL49R were presented. **D**–**E** A close-up of the zoomed area shows virions in the cytoplasm of BAC-CHv, BAC-CHv-ΔUL49, or BAC-CHv-UL49R infected cells. The nucleus (N) and cytoplasm **C** are marked.

VP22 can regulate the assembly of virus particles by interacting with many membrane proteins and glycoproteins [16]. Therefore, to explore the role of VP22 in the virus assembly process, we examined the ultrastructural morphology of BAC-CHv, BAC-CHv-ΔUL49 and BAC-CHv-UL49R viruses infected DEF cells by transmission electron microscopy. The cells infected by BAC-CHv, BAC-CHv-ΔUL49 and BAC-CHv-UL49R showed no nucleocapsids accumulation in the nucleus, but the cells infected by BAC-CHv-ΔUL49 showed obvious nucleocapsids accumulation in the cytoplasm (Figure 5). Only a few nucleocapsid gained the final envelope by membrane fusion, and the complete envelope was observed in both BAC-CHv and BAC-CHv- UL49R infection groups. Therefore, we speculated that VP22 affected the final envelope of the virus.

Furthermore, electron microscopy was used to observe the infected cells of three virus strains randomly, and 5 cells were selected for quantitative analysis of the virions at the secondary envelopment stage. Table 2 shows the percentage of virions during the secondary envelopment phase. In the cells which infected with BAC-CHv and BAC-CHv-UL49R, 73% and 74% of mature virus particles and only 27% and 26% of immature virus particles were found, respectively. At the same time, there were immature virus particles (66%) and mature virus particles (34%) in BAC-CHv-ΔUL49 infected cells, which were obviously different from BAC-CHv. The results manifested that DPV VP22 impaired the viral secondary envelopment and maturation.

**Table 2:** **A quantitative ultrastructural study on the second enveloping phase of DPV particles in DEF infected by virus.**

Virus	% particles by type		Total Counted (Virion /Cells)
	Mature virus	Immature virus	
BAC-CHv	73% (59)	27% (22)	81/5
BAC-CHv-ΔUL49	34% (33)	66% (64)	97/5
BAC-CHv-UL49R	74% (64)	26% (24)	88/5

Meanwhile, to understand the effect of DPV VP22 on the release of virions, we infected DEF cells with three viruses with MOI = 1 and cleaned the cell surface 3 times with PBS. After the new medium was replaced, the supernatant of cells was collected every 15 min. The number of infectious virions increased over time (Figure 4D), however, we found BAC-CHv-ΔUL49 showed significantly lower increasing level, which implying VP22 may inhibit virions release in vitro.

### VP22 significantly affects viral cell-to-cell spread

**Figure 6:**
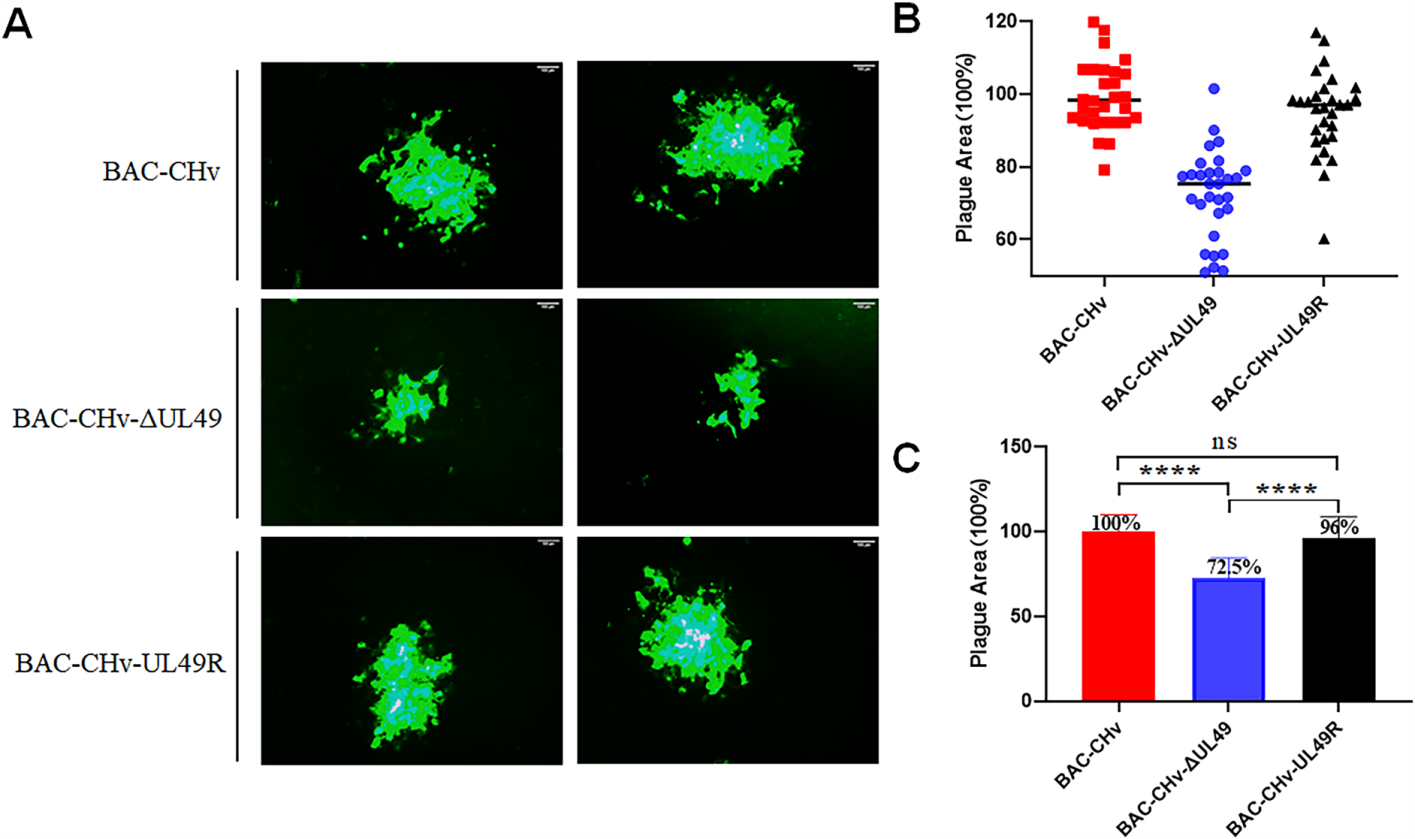
**The UL49 gene affects virus transmission between cells.** The recombinant viruses of BAC-CHv, BAC-CHv-ΔUL49 and BAC-CHv-UL49R were infected with 0.005 MOI, respectively. **A** Green fluorescent spots are produced by three recombinant viruses. **B–C** Thirty randomly selected plaques from each virus sample were measured and their areas were averaged relative to the BAC-CHv (****P < 0.0001).

To investigate the role of the DPV UL49 gene on the transmission between cells, we infected cells with 0.005-MOI virus and incubated it for 2 h. Then the medium was removed and cleaned the cell surface with PBS 3 times, and a medium (including 1.5% methylcellulose) was added to block the virion spread. We then detected the cell-to-cell spread of three viruses by surrogate the virus plague morphology with GFP expression captures under the microscope. We found that the plaque size generated by BAC-CHv-ΔUL49 was significantly smaller than that generated by BAC-CHv and BAC-CHv-UL49R, while the plaque size generated by BAC-CHv-UL49R was not significantly different from that of BAC-CHv (Figure 6A). To more directly assess the size of green fluorescent plaques, we randomly selected at least 30 green fluorescent plaques from each virus-infected cell for statistical analysis. As shown in Figures 6B, C, we regarded the size of the green fluorescent plaque of BAC-CHv as 100%, and found that the size of the fluorescent plaque of BAC-CHv-ΔUL49 was 72.5%, which decreased by 27.5% compared with that of BAC-CHv. The fluorescence plaque size of BAC-CHv-UL49R was 96%, which showed no significant difference compared with that of BAC-CHv, indicating that the plaque size generated by BAC-CHv-ΔUL49 significantly shrunk than that generated by BAC-CHv. These data suggest that deletion of the VP22 encoded by the DPV UL49 gene affects the spread of the virus from cell to cell.

### VP22 regulates viral mRNA accumulation

**Figure 7:**
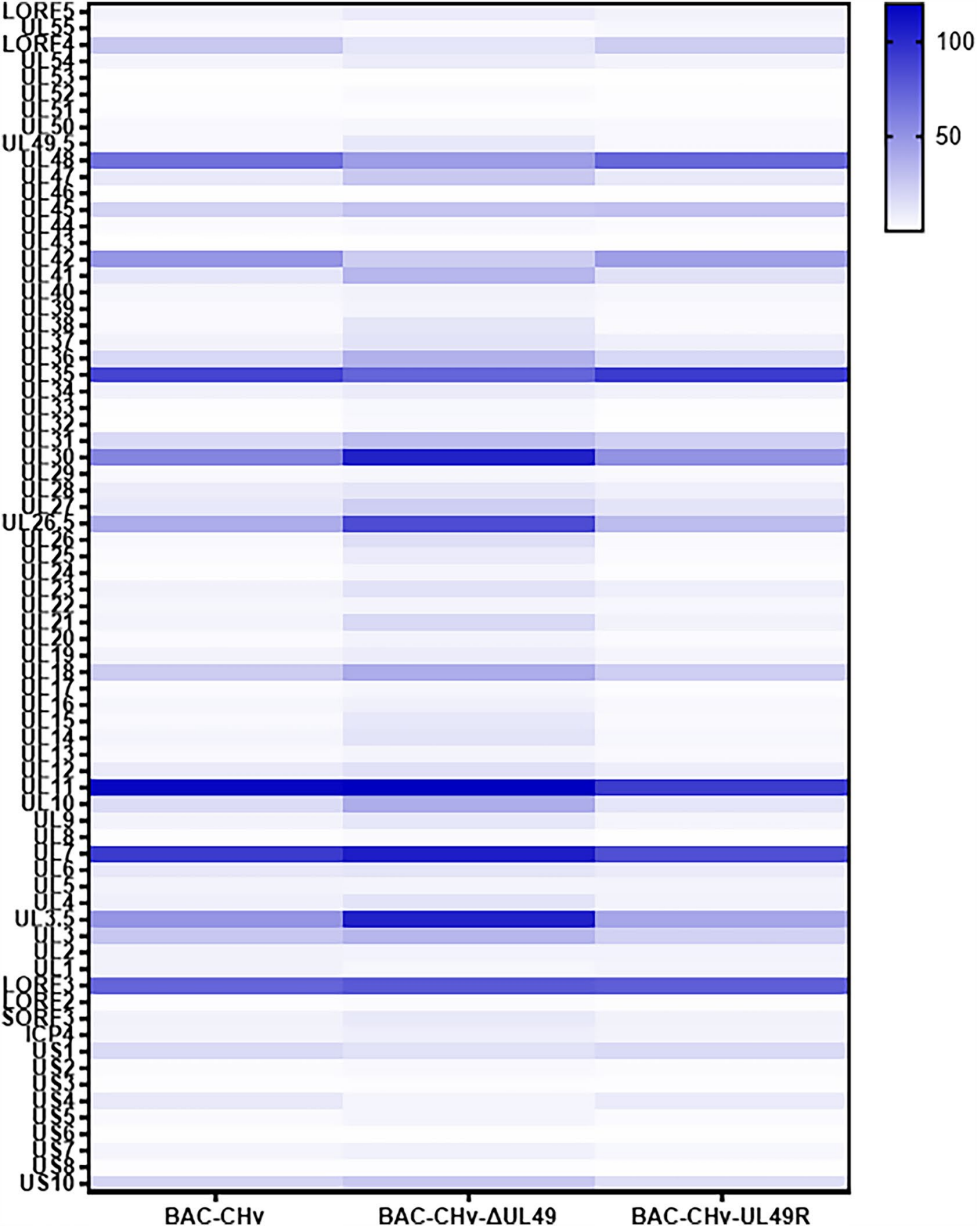
**The UL49 gene affects accumulation of viral mRNAs.** DEF cells were infected with recombinant viruses at an MOI of 1. Cells were harvested at 48 hpi, RNA was extracted from cells and RT-qPCR was used to detect mRNA levels. The relative gene expression levels were determined using the 2^−ΔCt^ method.

To further determine whether VP22 influences viral proliferation by regulating viral mRNA accumulation, we infected DEF cells with 1 MOI of the three viruses and collected cell samples at 48 hpi after infection. RNA was extracted from samples and real-time quantitative PCR (RT-qPCR) was used to detect mRNA levels of all DPV genes. As shown in Figure 7, the mRNA levels of most viral genes in BAC-CHv-ΔUL49-infected cells changed compared with BAC-CHv, but this change restored by the UL49 gene repair in the UL49 gene-deficient virus. These results illustrated that deletion of the UL49 gene significantly affects mRNA accumulation at the late stage of infection.

## Discussion

Based on the results of this study, we have drawn three important conclusions. First, DPV VP22 is a non-essential gene for virus replication, but it is essential for efficient virus growth in vitro; Second, deletion of VP22 affects the secondary envelopment of virus particles and the spread of virus particles between cells. Third, VP22 can regulate the accumulation of viral mRNA and thus affect the growth of the virus in DEF cells.

The product of UL49, VP22, is a highly abundant tegument protein with high expression in the virus infection [23]. Previous studies have shown that the ORF9 protein interacts directly with IE62, the major trans-activator of VZV, and co-localizes with microtubules, suggesting that the ORF9 protein may play a role in the late stages of infection [4]. Other observations also support this hypothesis: HSV-1 VP22 interacts with the tegument protein VP16 and the cellular microtubule network [24]. Here we showed that DPV VP22 is required for efficient DPV growth in cultured cells, but not for viral DNA replication. It is worth noting that EHV-1 VP22 is also not essential for pathogenicity in a hamster model, but is required for efficient viral growth in cultured cells [25]. In contrast, the VZV ORF9 protein and MDV VP22 are essential for productive viral replication in cell culture [26, 27]. Interestingly, PRV VP22 is dispensable for virus growth in vitro and has no effect on virulence and neuronal spread in rodents [28]. A possible explanation for this might be that the effect of viral proteins on viral replication is related to the type of virus.

Importantly, although much of the literature reports a role for VP22, and its homologues, in the envelopment, the molecular mechanisms and interactions that support these key steps remain poorly understood. In HSV-1 infected cells, VP22 interacted with many viral proteins including UL11, UL16, UL21, and gE, suggesting that VP22 may form a protein scaffold with other tegument proteins and provide a protein bridge between the envelope and capsid, this will facilitate protein packaging into the virus particles [29, 30]. Through ultrastructural analysis, we found that the absence of DPV VP22 results in a large number of nucleocapsids clustered around intracellular vesicles, with few outgrowths into the vesicles, which has a significant impact on the secondary envelopment of the viral particles. Thus, we speculate that the deletion of VP22 not only disrupts its bridging function in the secondary envelopment phase, but may also result in other proteins not reaching the correct location. However, it is possible that other tegument proteins exist in the virus to compensate for the missing role of VP22 and are used to form a stability protein network that the tegument proteins-glycoproteins and thus allows the budding process to take place, which warranted future investigation.

All herpesviruses are cell-to-cell transmissible by delivering mature viral particles containing cytoplasmic vesicles to the cell junction so that they can enter adjacent uninfected cells, and also avoids the antiviral capacity of neutralizing antibodies [31]. The homologue of VP22 has the unusual property of efficient intercellular transport. For example, HSV-1 VP22 is associated with an actin-associated motor protein (NMIIA), and this interaction may be associated with viral transport or export [32]. The HSV-1 VP22 deletion mutant significantly reduced virus release in cultured cells [33]. Cell-to-cell spread facilitates rapid virus spread and may also evade host immune surveillance [10]. Meanwhile, HSV-1 VP22 evades cGAS/STING-mediated innate immunity by inhibiting cGAS enzyme activity [34]. HSV-1 VP22 have been reported to be transported between cells and transmits nucleic acids and proteins to unprepared and unintentional cells [35]. We found that DPV VP22 is important for the release of the virus and its transport between cells. Because the innate immune system of duck and human is different [36], we suggested that the mechanism of DPV VP22 is different from HSV-1 VP22 in the escape of the innate immune response. Based on the above phenomena, we therefore speculate that DPV VP22 may evade host immune surveillance by transporting transmits nucleic acids and proteins between cells, thereby suppressing innate immune response.

Moreover, the proteins encoded by the HSV-1 UL47, UL49, and US11 can bind to cell mRNA in host cells, and packaged RNA can be expressed in infected cells. VP22 can be used as a carrier of messenger RNA to mediate the transfer of RNA from infected to uninfected cells, thereby creating an environment for initiating an effective infection [37]. An early study showed that deletion of HSV-1 VP22 result in a small decrease in mRNA levels and a defect in mRNA abundance-independent multimeric assembly, both of which can be complemented by secondary mutations in virion host shutoff (VHS) [20]. DPV VHS has been shown to influence viral replication by regulating mRNA levels [38]. HSV-1 VP22 promotes the accumulation of viral mRNA in the early stage of viral infection and the synthesis of protein in the late stage of viral infection [39]. Here, we found that DPV VP22 influenced viral mRNA accumulation and hypothesized that DPV VP22 affects viral growth by regulating mRNA levels. We speculate that the main limitation is that we did not detect mRNA levels from early to late after infection VP22-deficient viruses, but only at the 48-h time point after viral infection, and therefore we were unable to determine at what time of viral infection VP22 promotes mRNA accumulation, and despite the limitations, this study can clearly indicate that VP22 deficiency affects viral mRNA accumulation.

In conclusion, this study found that VP22 is required for the efficient growth of DPV in cultured cells in vitro. Meanwhile, VP22 plays a key role in the life cycle by regulating the secondary envelopment, cell-to-cell spread, release, and the accumulation of viral RNA. Our findings provide insights into the function of DPV VP22 and provide detailed information for the prevention and treatment of DPV.

## Supplementary Information

**Additional file 1. Sequences and primer pair characteristics**.
